# Recent advances in understanding the role of the hypothalamic circuit during aggression

**DOI:** 10.3389/fnsys.2014.00168

**Published:** 2014-09-25

**Authors:** Annegret L. Falkner, Dayu Lin

**Affiliations:** ^1^Neuroscience Institute, New York University School of MedicineNew York, NY, USA; ^2^Department of Psychiatry, New York University School of MedicineNew York, NY, USA

**Keywords:** aggression, hypothalmus, VMHvl, optogenetic stimulation, circuits, estrogen receptor alpha, aggressive motivation, optogenetics

## Abstract

The hypothalamus was first implicated in the classic “fight or flight” response nearly a century ago, and since then, many important strides have been made in understanding both the circuitry and the neural dynamics underlying the generation of these behaviors. In this review, we will focus on the role of the hypothalamus in aggression, paying particular attention to recent advances in the field that have allowed for functional identification of relevant hypothalamic subnuclei. Recent progress in this field has been aided by the development of new techniques for functional manipulation including optogenetics and pharmacogenetics, as well as advances in technology used for chronic *in vivo* recordings during complex social behaviors. We will examine the role of the hypothalamus through the complimentary lenses of (1) loss of function studies, including pharmacology and pharmacogenetics; (2) gain of function studies, including specific comparisons between results from classic electrical stimulation studies and more recent work using optogenetics; and (3) neural activity, including both immediate early gene and awake-behaving recordings. Lastly, we will outline current approaches to identifying the precise role of the hypothalamus in promoting aggressive motivation and aggressive action.

Aggression is a primary social behavior used by humans and animals alike to defend territory, secure mates, compete for food, and protect young. The term aggression comes from the Latin word *aggressio*, meaning to attack, but also to approach and initiate action. Specific actions associated with aggression depend on the species, but can include biting, kicking, hitting or pushing. In addition, threatening displays such as hisses, enlarged body size, and changes in facial expression are also parts of the aggressive repertoire of many species. Although humans express aggression in diverse physical and verbal forms (often separated into “reactive” or “instrumental” aggressive behaviors), the underlying goal of human aggression—defense and competition for resources—remains the same. Increases in both human and animal aggression are accompanied by similar autonomic responses including raised heart rate and respiration, and both are influenced by changes in circulating hormones, such as testosterone (Nelson and Trainor, 2007). While the specific actions and musculature used during aggression may differ between humans and animals, the underlying neural mechanisms that drive aggressive behavior are likely to be largely conserved across species.

For nearly a century, neuroscientists have sought to understand the neural basis of aggression by perturbing and monitoring brain activity through a variety of methods. Numerous classic lesion and electric stimulation experiments have established the hypothalamus as a crucial node for the expression of aggressive behavior (Clemente and Chase, 1973; Siegel et al., 1999), but its role in promoting these behaviors has remained elusive. Newly emerging techniques for measuring and manipulating neural circuitry, including optogenetic and pharmacogenetic tools and advances in technology for *in vivo* recording and imaging in the freely moving animal, have opened new avenues for research on aggression and allow study at the level of genetically defined cell type and the single neuron. In this review, we will focus on this recent progress and provide an up-to-date view on the role of the hypothalamus in promoting aggression and compare this to its role in promoting other social interactions such as sexual behavior. Since these novel genetic-based functional manipulation tools are most powerfully applied in the laboratory mouse, this review will focus mainly on advances in understanding the neural substrates of rodent aggression. However, novel contributions to our understanding of aggression circuitry are also currently being done in non-rodent species including humans (Goodson et al., 2012; Franzini et al., 2013; Haller, 2013; Torres et al., 2013).

## Novel approaches to functional manipulation of hypothalamic circuitry

### Targeted hypothalamic inactivation reduces natural inter-male aggression

Many studies have attempted to assess the role of the hypothalamus through inactivation experiments, using diverse surgical, pharmacological, and genetic methods. Some of the earliest experiments on aggression were knife cut experiments performed on cats during the 1920’s which demonstrated the importance of hypothalamus in the expression of rage (Bard, 1928). When the forebrain area was dissociated from its posterior structures, leaving the hypothalamus and its downstream connections intact, operated cats showed spontaneous and unprovoked aggressive behaviors (which they termed *sham rage*), such as hissing and paw striking. In contrast, if the cut was made posterior to the caudal hypothalamus, these behaviors were absent, indicating that an intact hypothalamus is indispensable for the generation of these rage behaviors.

Further experiments demonstrated that specific subnuclei within the hypothalamus may be preferentially involved in promoting aggression, though the precise effects remained unclear. Contradictory results were reported regarding the effects of electrolytic lesions of the medial hypothalamus on aggression: while some reports showed decreased aggression after lesioning, others showed the opposite trend (Grossman, 1972; Olivier and Wiepkema, 1974; Oliver, 1977; Albert and Walsh, 1982; Albert et al., 1987). Reasons for these inconsistent results may be the poor spatial control of the lesion site, damage to fibers of passage and/or post-operation compensation. In addition, pharmacological manipulations have provided additional evidence supporting the necessary role for specific hypothalamic subnuclei in natural aggression: blockage of substance P receptor in medial hypothalamus or the destruction of substance P expressing neurons lowers the number of “violent” hard bites in rats (Halasz et al., 2008, 2009) and injection of vasopressin receptor antagonist into the anterior hypothalamic nucleus (AHN) of hamster decreased the number of attacks and increased attack latency (Ferris and Potegal, 1988).

**Figure 1:**
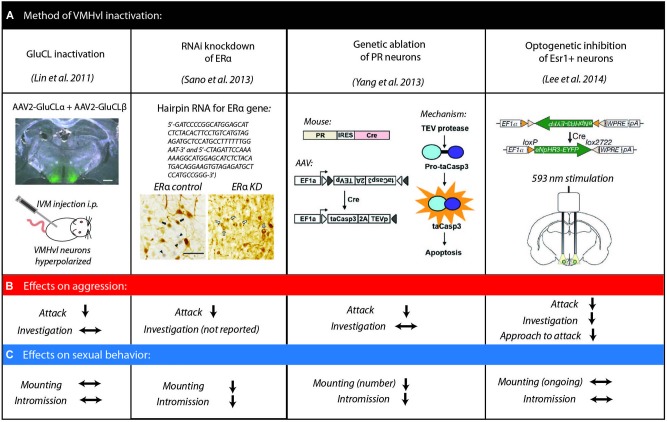
**Methods of inactivation of hypothalamic VMHvl neurons to examine fighting and mating behavior. (A)** Summary of methods adapted from Lin et al. (2011); Sano et al. (2013); Yang et al. (2013); Lee et al. (2014) with permissions. Effects on aggressive behaviors **(B)** and sexual behaviors **(C)** for each inactivation method.

More recent work has focused on a smaller subdivision of the hypothalamus in mouse, the ventromedial hypothalamus, ventrolateral part (VMHvl), and these results have provided the most clear picture to date of the role of the hypothalamus in aggression though its role in mating behavior is less well understood (Figure 1). Several novel approaches have been taken to reduce activity in the VMHvl (Figure 1A). For example, we reversibly inhibited the VMHvl using virally expressed *Caenorhabditis elegans* ivermectin (IVM)-gated chloride channel (GluCL), which prevents the initiation of action potentials by hyperpolarizing the cells upon ligand binding. We found that attack, but not intermale social investigation, is strongly suppressed by VMHvl inhibition, and this decreased aggression returns to normal levels when VMHvl activity is restored (Lin et al., 2011).

Other studies have capitalized on the fact that the VMHvl is enriched in estrogen receptor alpha (ERα). Since ERα knockout mice exhibit a severe reduction in aggression, these studies examined the role of ERα cells within the VMHvl on male mouse aggression (Ogawa et al., 1997; Merchenthaler et al., 2004). Both RNAi knockdown of ERα in the VMHvl and ablation of VMHvl cells expressing progesterone receptor (PR), which largely overlaps with the ERα neuronal population, resulted in a dramatic decrease of natural inter-male aggression (Sano et al., 2013; Yang et al., 2013). Most recently, optogenetic inhibition of VMHvl ERα cells during both social approach and attack showed that ongoing activity of VMHvl ERα cells is necessary for both initiation and continuation of attack (Lee et al., 2014).

While the effects of these genetically targeted VMHvl manipulation studies on intermale aggression show clear suppression of attack behaviors, the effects on mating were more varied (Figure 1C). When ERα expression is suppressed or PR cells are ablated in the VMHvl, male sexual behavior, like aggression, is reduced. Specifically, manipulated animals spent less time intromitting and achieved fewer ejaculations although the number of mount episodes was not affected (Sano et al., 2013; Yang et al., 2013). In contrast, acute optogenetic inhibition of ERα cells in the VMHvl or pharmacogenetic inhibition of VMHvl in a genetically nonselective manner does not disrupt ongoing sexual behaviors (Lee et al., 2014). These conflicting data suggest that the VMHvl may promote the advance of sexual behaviors but be indispensible for the initiation and execution of attack.

### Hypothalamic activation induces attack in mouse

The flipside of the loss-of-function or inactivation study is to artificially activate targeted brain regions and examine the resultant behavioral changes. Electric stimulation of the hypothalamus has been reported to induce attack in a variety of mammalian species including rat, cat and monkey (Lipp, 1978; Lammers et al., 1988; Siegel and Pott, 1988; Siegel et al., 1999). However, electric current activates not only cell bodies but also fibers of passage, thus limiting the control and identification of the neurons responsible for any observed behavioral changes. In comparison, more recent optogenetic approaches have allowed experimenters to target locations within the hypothalamus with higher precision and to identify the targeted cells with more confidence. Advances in location targeting using optogenetic techniques are particularly evident when using mice as a model organism, whose brains are small in comparison to rats or cats. For example, in mice the VMHvl spans only 700 μm along the anterior-posterior axis, 400 μm medial-laterally and 200–400 μm dorsal-ventrally depending on the anterior-posterior position, making this subnuclei only a quarter of the volume of the VMHvl in rats and difficult to isolate with electrical stimulation.

**Figure 2:**
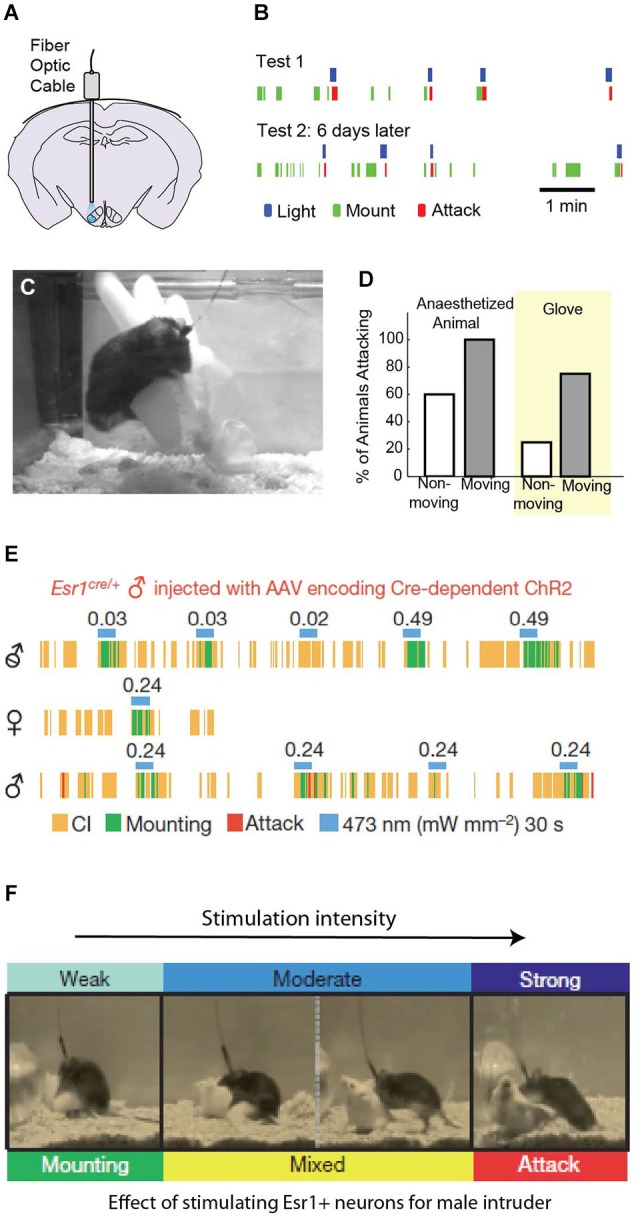
**Optogenetic activation of VMHvl neurons evokes attack.** Stimulation of wild type VMHvl neurons **(A)** reliably evokes attack to a female or castrated male “intruder” **(B)** and also to either an anesthetized mouse or inanimate object **(C–D)**. **(A–D)** Adapted from Lin et al. (2011) with permissions. Activation of a targeted subpopulation of ERα neurons in the VMHvl evokes mounting at low intensity **(E)**, and attack at high stimulation intensity **(F)** while moderate stimulation evokes a mix of aggressive and sexual behaviors (**E–F** adapted from Lee et al., 2014 with permissions).

To activate neurons using optogenetic methods, a light gated cation channel, channelrhodopsin (ChR2), often fused with fluorescent protein, is virally or genetically expressed in a specific brain region or a group of molecularly defined cells. Following expression, light pulses delivered through an implanted optic fiber (Figure 2A) can control the spiking activity of ChR2 expressing cells with high temporal precision (Boyden et al., 2005). Using this method, we induced attack in mice from a nonselectively infected group of cells in VMHvl (Figures 2B–D). In contrast, electric stimulation of the VMHvl in mice evoked defensive-like responses and could even cause disengagement during ongoing fighting. These differences in electrically stimulated behaviors are likely due to the activation of axons from the adjacent dorsomedial and central VMH during stimulation which form a part of the defense circuit (Martinez et al., 2008). Although attack can be electrically elicited in rats from a continuous functionally defined “hypothalamic attack area (HAA)” covering VMHvl and its lateral and anterior areas, optogenetic stimulation in mice induces attack only when ChR2 is expressed at least partially in the VMHvl, suggesting that cells in the VMHvl but not its surrounding area are crucial for stimulation-induced attack (Lammers et al., 1988; Lin et al., 2011). This conclusion is further substantiated by restricting ChR2 expression using ERα-CRE transgenic mouse. Activating estrogen receptor-expressing VMHvl cells elicits attack towards males, castrated males, and females (Figure 2E), whereas activating estrogen non-expressing cells (“CRE-out”) produced no increase in attack (Lee et al., 2014). Taken together, VMHvl has been established as a key hypothalamic node in mediating male mouse aggression. Further experiments which look specifically at the responses of estrogen receptor-expressing neurons during natural behavior, and also at the role of PR expressing neurons will refine our understanding of the role of the VMHvl.

### Hypothalamic neural substrates for female aggression

In most species, males are significantly more aggressive than females, and the vast majority of studies have focused on the role of the VMHvl in intermale aggression. However, although the VMHvl is anatomically sexually dimorphic, with males possessing a larger neural volume, several studies have probed whether there is an analogous hypothalamic substrate for female aggression (Matsumoto and Arai, 1986). Electric stimulation of the hypothalamus in female rats suggests that a functionally defined HAA also exists in the female brain that spans approximately the same area as it does in males (Kruk et al., 1984). More recently, optogenetic activation of the VMHvl ERα subpopulation in female mice failed to elicit attack and instead caused stimulated females to exhibit male-style mounting behaviors (Lee et al., 2014). While these results may suggest that different neural substrates mediate female aggression vs. male aggression in mice, several alternative explanations remain possible. First, in females, aggression may be mediated by non-ERα cells or anatomically distinct neurons. Instead, ERα cells in VMHvl are critical for female sexual behavior: suppressing ERα expression in the VMHvl using RNAi or deleting PR expressing cells in VMHvl significantly reduces receptivity of manipulated animals (Sano et al., 2013; Yang et al., 2013). Second, failure to evoke aggression may be related to the specific animals used in these experiments, since natural aggression levels of female mice or rats differs dramatically between laboratory strains. For instance, the CPB WEzob female rats used for electric stimulation present high level of aggression towards intruders even during non-lactating periods, whereas the genetic background of ERα-CRE mice is C57BL/6 which exhibit little aggression even during lactation (Broida and Svare, 1982; Jones and Brain, 1987). It remains possible that the neural substrates of aggression are abnormally suppressed or degenerated in female C57BL/6 mice, which may make it difficult to elicit attack with artificial activation. Future experiments using mouse strains with higher female aggression levels may help explain the observed behavioral difference in female rats and mice during hypothalamic stimulation.

### Interpreting stimulation-evoked attack: insights from rodent behavior

First asked by Hess (1928) almost a century ago, the question of how we should interpret the effects of stimulation on aggression still remains unresolved. It is tempting to posit the role of the hypothalamus in aggression as a simple “attack generator”, but such descriptions do not account for the complexity of stimulation-evoked responses. To start with, both earlier electric stimulation and more recent optogenetic activation experiments demonstrate that hypothalamic evoked attack depends on the presence and reactions of an attack target. When a resident mouse is alone in its home cage (with no target to direct an attack towards), light stimulation induces only slight increases in locomotion, and animals do not exhibit any of the motor patterns typical of aggression (Lin et al., 2011). Similarly, electric stimulation of HAA in rats produces no motor change although it does cause fast adrenocortical stress responses even in the absence of an opponent (Kruk et al., 2004).

In both rats and mice, hypothalamic activation induced attack is also affected by opponents’ variable defensive tactics. For instance, if an opponent’s back is against the wall, stimulated mice often abort the evoked attack, possibly in part because the preferred biting location is not immediately accessible. In rats, the mode of attack depends critically on the response and location of the opponent: attack jumps arise when the opponents go into an upright position whereas so-called “clinch fights” occur when one of the rats loses balance following an attack jump (Kruk et al., 1983). Because the specific actions evoked by stimulation depend on the context of the fight, the effects of stimulation are unlikely to be that of a stereotyped motor response. Consistent with this conclusion, neither VMHvl nor HAA has direct connection with known voluntary motor coordination structures, such as the basal ganglia (Canteras et al., 1994; Roeling et al., 1994). However, while stimulation-induced attack appears to require a target, the identity of the target may be suboptimal. In both rats and mice, hypothalamic stimulation can elicit quick attack towards anesthetized or dead conspecifics, potential mates, or certain inanimate objects (like an inflated rubber glove), all of which evoke no attack naturally (Koolhaas, 1978; Kruk et al., 1979; Lin et al., 2011). Thus, hypothalamic stimulation induced attack appears to provide a fictive signal that is independent of social context. One possibility is that hypothalamic stimulation increases the animal’s predisposition or motivation to attack, while the specific movements that comprise the attack itself are encoded in downstream areas. This is discussed later in this review.

### Hypothalamic control of innate behavior

How are aggression-related neurons organized in relation to neurons mediating other innate behaviors? Answers to this question have evolved over time as activation techniques have become increasingly refined. In previous studies, it was discovered that radically different behaviors could be evoked by electrically stimulating from the same electrode tip if one varied either the stimulation duration, intensity, frequency, or experimental environment (Valenstein et al., 1968; Watson, 1977; Kruk, 2014). The lack of a stable behavioral response from a given anatomical location led researchers to initially conclude that there was no fixed association between specific hypothalamic neural substrates and individual behavioral outputs. As recording and stimulation technologies improved, the size of the stimulation electrodes decreased such that anatomical locations could be targeted with greater precision. Systematic mapping efforts with these electrodes revealed that predictable behaviors could be evoked from specific coordinates, demonstrating that neural substrates underlying behaviors are not diffusively present throughout the hypothalamus (Kruk et al., 1983; Lammers et al., 1988). However, even with these technical refinements, multiple behaviors could often be elicited from the same area across changing stimulation intensity.

Given this overlap of behaviors evoked within a single stimulation site, the question remains whether discrete sets of neurons trigger specific patterns of behavior, or whether behavioral states are encoded by complex patterns of activation in overlapping neural circuits. More directly, do changes in evoked behaviors over stimulation intensity reflect an “intensity coding” in the hypothalamus or a limitation in cell targeting? Recent results using both optogenetics and imaging techniques suggest that the answer appears to be both. For some co-elicited yet distinct behaviors, such as flight and attack, targeted optogenetic stimulation experiments demonstrate that these behaviors likely involve non-overlapping but adjacent hypothalamic areas (Lin et al., 2011). Other behaviors that are commonly co-elicited by electrical stimulation, including social grooming, investigation and attack, can be similarly evoked with optogenetic stimulation: low intensity stimulation induces forceful investigation (sometimes called “close investigation”) of the intruder while higher intensity stimulation can elicit attack (Lee et al., 2014), though these behaviors may sometimes be considered part of the aggressive repertoire.

Surprisingly, although mounting has never been reported to be elicited from HAA using electrical stimulation, optogenetic activation in mice has revealed that mounting and fighting can be induced by activating ERα neurons in the VMHvl at different light intensity: low intensity stimulation induces mounting towards both females, males, and castrated males, while higher light intensity evokes mixed attack, with mounting initially and eventually attack exclusively (Figure 2F; Lee et al., 2014), Whether the same ERα neurons are involved in mediating both mating and fighting (which would be strong evidence for intensity coding) is not yet known, as ERα neuron activity during mating and/or fighting has not been reported. However, extracellular recordings from non-selective cells in the VMHvl reveals no simple relationship between VMHvl cell responses to males and females (See next section). VMHvl activity escalates as animals switch from male investigation to attack but decreases as animals switch from female investigation to mounting (Lin et al., 2011). Optogenetic activation of a subset of ERα cells at a fixed rate and intensity is unlikely to faithfully recapture the natural activity progression during female or male interaction, and instead, low intensity activation may more closely resemble the female conspecific induced response, while high intensity activation may resemble a more male-like response pattern.

## Neuronal activity in the hypothalamus

A full understanding of hypothalamic functioning during complex social behaviors such as aggression will require a complete description of changing neural activity obtained during well-controlled social environments. However, most of what we know about neural activity during aggression is a crude proxy based on changes in either metabolic level (e.g., 2-deoxyglucose mapping and functional magnetic resonance imaging (fMRI)) or from the expression of immediate early genes (IEGs) such as Fos (Sagar et al., 1988; Kollack-Walker and Newman, 1995; Kollack-Walker et al., 1997; Delville et al., 2000; Gammie and Nelson, 2001; Hasen and Gammie, 2005, 2006; Veening et al., 2005; Haller et al., 2006; Ferris et al., 2008). Across all species and all social behaviors, only a handful of studies have attempted to directly link spiking activity and behaviors by recording populations of hypothalamic neurons in awake behaving animals (Oomura et al., 1983, 1988; Aou et al., 1984, 1988; Horio et al., 1986; Shimura et al., 1994; Lin et al., 2011; Falkner et al., 2014), yet the results from these studies shed considerable light on hypothalamic functioning.

### Immediate early genes and the hypothalamus during aggression

The induction of IEGs, in particular Fos, has been of great use throughout the last few decades in mapping brain-wide patterns of activation for both neural and endocrinological signals (Sagar et al., 1988; Bullitt, 1990; Sheng and Greenberg, 1990; Hoffman et al., 1993). Since the time course of IEG induction is relatively slow (5–30 min for IEG mRNA and 1–2 h for IEG protein), it is incapable of reporting detailed correlation between specific behavioral events (e.g., attack) and neural activity. Instead, IEG expression gives a general impression of accumulated brain activation over minutes to hours. Furthermore, the relationship between IEG induction and cell spiking activities remains uncertain. The most popular IEG—Fos, is a transcription factor induced by elevated cAMP levels and is not necessarily associated with action potential increases (Metz and Ziff, 1991). Nevertheless, these experiments are useful in that they provide a comprehensive view of candidate brain regions involved in a behavior. Moreover, examining patterns of co-expression of IEGs and other genetic markers or tracers can provide additional information regarding features of the behaviorally relevant neural population, such as their neurotransmitter type or projection pattern.

**Figure 3:**
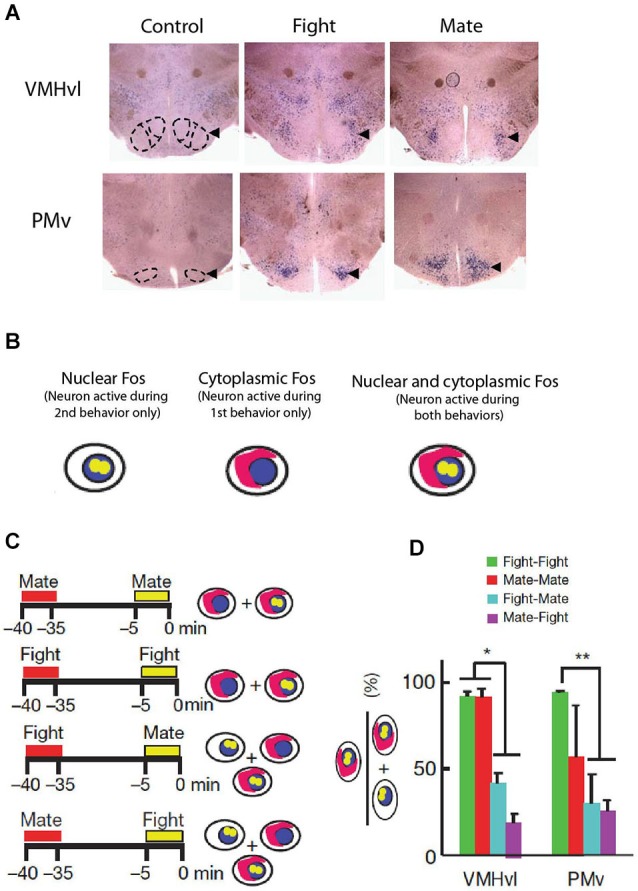
**Immediate early gene induction in hypothalamic neurons during fighting and mating behaviors. (A)** Traditional Fos induction paradigms show increased activation during fighting and mating in the VMHvl and PMv but do not reveal whether their activation patterns overlap. **(B–C)** Methodology of Fos CatFish paradigm, which allows for localization of neurons involved in both behaviors (fight-mate and mate fight) in comparison to the reliability of a single behavior (mate-mate and fight-fight). **(D)** Fos CatFish induction reveals largely non-overlapping hypothalamic populations activated during fighting and mating. **(A–D** adapted from Lin et al., 2011 with permissions).

A typical IEG induction paradigm for the study of aggression is the “resident-intruder” assay. In this paradigm, a male intruder is introduced into the home cage of a singly housed male conspecific for 5 min to an hour, which typically elicits repeated investigative and attack behaviors from the aggressive resident towards the intruder. Across rodent species, this resident-intruder test induces elevated Fos expression in several hypothalamic nuclei, including the medial preoptic nucleus (MPN), AHN, VMHvl and premammillary nucleus ventral part (PMv; Figure 3A; Kollack-Walker and Newman, 1995; Delville et al., 2000; Halasz et al., 2002a; Veening et al., 2005; Haller et al., 2006). The increase in Fos-expressing neurons following induction ranges from 50% to 300% of the baseline activation level. Across many IEG studies, the co-activation of these four hypothalamic nuclei during the resident intruder assay is consistent with their patterns of connectivity. Since the MPN, VMHvl and PMv are reciprocally interconnected and all receive inputs from the medial amygdala subdivision responding to female cues, they have also been proposed as the hypothalamic reproductive circuit (Swanson, 2000). Although AHN is traditionally included in the hypothalamic defense circuit, it receives moderate input from the VMHvl and represents the only major connection between the reproductive and defense hypothalamic circuits and so may place a more specialized role in aggression (Canteras et al., 1994).

Most Fos induction studies also attempt to examine the specificity of the region in mediating aggression by comparing Fos induction after aggression to the Fos activation pattern after a related behavior. For example, Delville et al. (2000) compared Fos expression of hamsters exposed to a woodblock carrying intruder odor and those exposed to an alive intruder and noticed increased Fos in the VMHvl after fighting, supporting a role of VMHvl in consummatory aspect of aggression and not simply that of sensory relay for male social odor cues (Delville et al., 2000). However, comparing activation patterns between two behaviors between different animals cannot reveal whether the same or different cells are involved, making it sometimes difficult to interpret these results. For example, Kollack-Walker et al. reported that PMv and VMHvl are activated similarly after both fighting and mating, though it was unclear whether these activated neurons represented overlapping or non-overlapping populations (Kollack-Walker and Newman, 1995). Similarly, defeated animals who were on the receiving end of attack showed stronger Fos expression in VMHvl than the attackers, even though activating VMHvl induces attack itself (Kollack-Walker et al., 1997). Do these extra neurons in defeated neurons represent a separately activated population of neurons?

These results demonstrate the necessity of comparing IEG activation patterns following two behaviors within the same animal. Since IEGs are first transcribed in the nucleus and then (after ~30 min) translocate to the cytoplasm, the IEG pattern associated with two properly spaced behaviors can be distinguished based on mRNA localization (Figure 3B; Guzowski et al., 2001). This method, named Cellular Compartment Analysis of Temporal Activity by Fluorescence *In Situ* Hybridization (CatFish), revealed importantly that mating and fighting induced Fos in male mice is mostly localized in intermingled but non-overlapping cells throughout the hypothalamus (Figures 3C,D; Lin et al., 2011). Recently, several other genetic methods that permanently mark IEG expressing cells in a short window have also been described (Reijmers et al., 2007; Guenthner et al., 2013; Kawashima et al., 2013); these methods can be potentially of great use in future studies to address the relationship between neural population(s) involved in aggression and other related behaviors.

### Neural recording in the hypothalamus

Perhaps because of its heterogeneity, small size and deep position in the brain, the hypothalamus has long resisted physiological scrutiny. In particular, *in vivo* recordings performed during ethologically relevant behaviors (e.g., fighting) have been difficult because these behaviors often involve brief violent bursts of action that are not ideal for conducting stable recordings, and also because previous recording technologies were too cumbersome for animals to perform complex, quick movement with these devices attached to their heads. Fortunately, recent advances in both the stability and size of chronic extracellular recording technology have allowed us to begin interrogating these nuclei during behaviors of interest.

**Figure 4:**
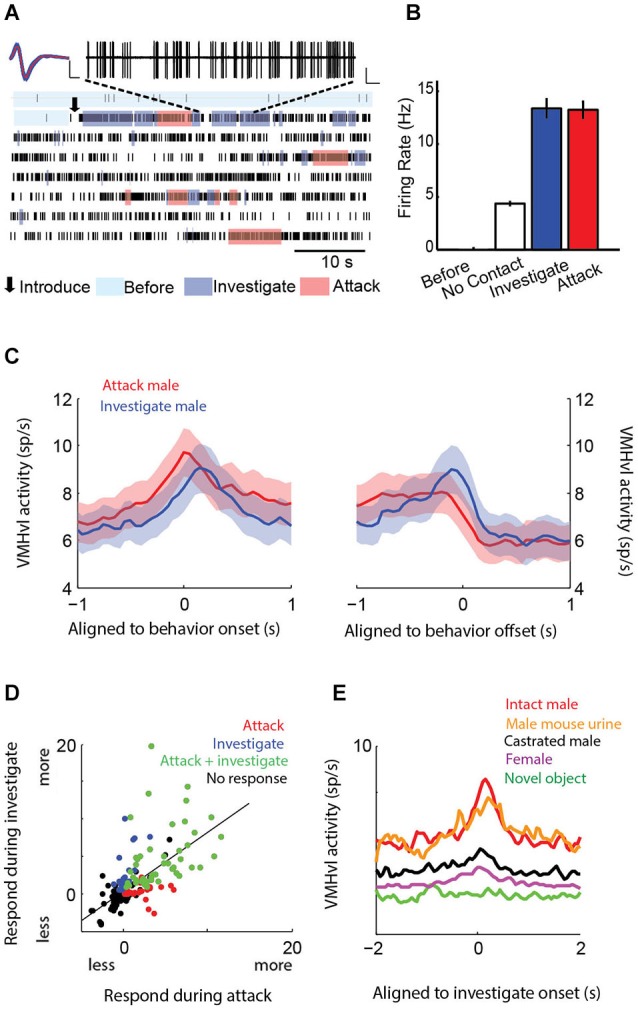
**Neural recordings of hypothalamic neurons show activation during attack and investigation of a male intruder**. Single neurons **(A–B)** and population average peri-event time histogram **(C)** exhibit acute increases in activity at the onset of both attack and investigation. **(D)** Individual neurons can show selectivity for attack (red), investigation (blue), both behaviors (green), or no increase during either of those behaviors (black). **(E)** VMHvl neurons are activated during the investigation of a source of male mouse urine, but activated little by a castrated male, female, or novel object. **(A–B** adapted from Lin et al., 2011; **C–E** adapted from Falkner et al., 2014 with permissions).

We performed chronic *in vivo* recordings of populations of VMHvl neurons during fighting and mating (Lin et al., 2011) and compared these responses to a variety of interactions with social and nonsocial stimuli including a castrated male, a source of either male and female urine, and a novel object (Falkner et al., 2014). Consistent with the results of functional manipulation studies, over 40% of VMHvl cells increase their activity when the recorded mice encounter a male intruder (Figures 4A–C). VMHvl activity begins to increase several seconds prior to attack initiation and reaches its peak at the onset of attack. Activity then persists at an elevated level for the duration of attack, plummeting when attack ceases (Figure 4C). Like attack, neurons also acutely increase in activity during the investigation of a male intruder and these responses are highly correlated: neurons that exhibit increased activity during attack are also likely to increase during investigation (Figure 4D). While these responses are correlated across the population, individual neurons may respond selectively during attack (red dots Figure 4D), during investigation (blue dots Figure 4D), or during both behaviors (green dots Figure 4D) such that they may be preferentially active during either the sensory acquisition or the action phase of aggression. Black dots in Figure 4D represent neurons that increase neither during attack nor investigation.

Similar to social behaviors, olfactory cues alone appear to be a strong driving force to hypothalamic activity. VMHvl neurons increase their firing rate during the investigation of a source of male mouse urine to similar levels as when the animal investigates a male conspecific (Figure 4E). Activity is inversely correlated with either the distance from the male intruder or source of urine. In contrast, investigation of castrated males, who produce reduced levels of social olfactory cues, evoked little increase in VMHvl neuron activity, demonstrating that the odor cue may be more important than the presence of a social stimulus in driving hypothalamic activity (Figure 4E).

**Figure 5:**
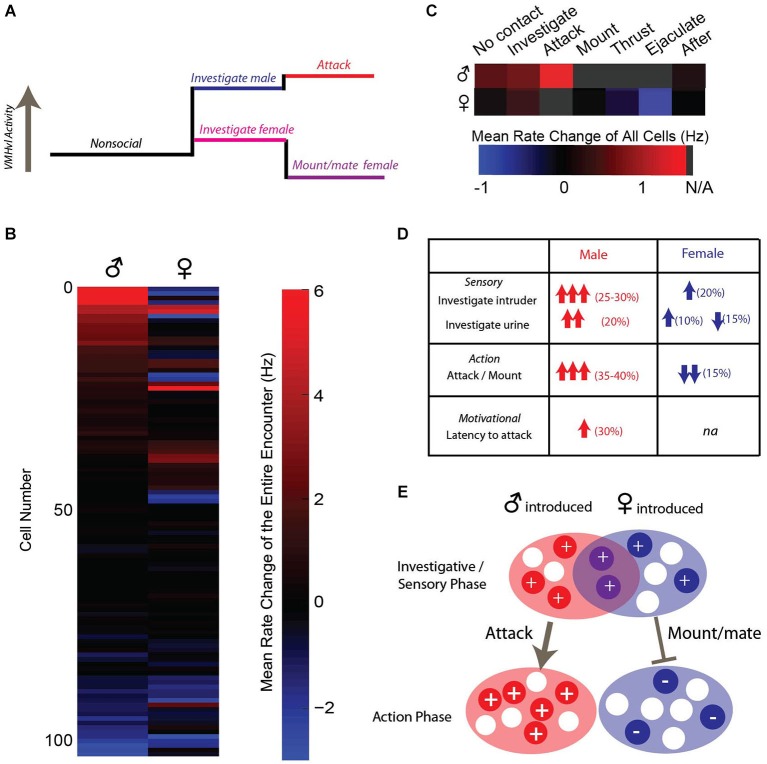
**VMHvl activity during male and female interactions reveals opposing response patterns. (A)** Average activity during a male interaction increases during investigation and attack, while average activity increases slightly during investigation of a female and is suppressed during subsequent sexual behaviors. **(B)** Response profile across VMHvl neurons during social interactions suggests that neurons that increase activity during male interactions may be suppressed during female interactions but not vice versa. **(C–D)** Summary of VMHvl neurons participating in separable phases of social interactions. **(E)** Schematic illustrating that neurons may increase in activity during the sensory acquisition phase of social behavior to both sexes, while the action phase of the social interaction results in opposing activation patterns in populations with less overlap. **(B–C** adapted from Lin et al., 2011 with permissions).

Neural recordings can begin to directly address how signals for fighting and mating are intermingled within populations of hypothalamic neurons. Unlike IEG studies, which do not have the temporal resolution to address differences between the investigative phase and the action phase (attack or mount), physiological activity in the VMHvl shows clear differences in activity during separable phases of male and female interactions (Figure 5A). During interactions with an intruder male, a substantial subpopulation shows an increase during investigation of both a male and a male social odor, and this can be followed by a transition into a greater degree of activation during attack (Figures 5B–D). In contrast, responses during the investigation of a female or a female social odor cue are more mixed, engaging a smaller subpopulation of neurons, with a higher portion of them exhibiting suppressive responses during the investigation. The action phase of an encounter with a female (mounting and subsequent mating behaviors) results in a decrease or suppression of overall activity, the opposite of the action phase during intermale encounters (Figures 5C–E). This leaves open the critical yet unanswered question of how stimulation (which should increase activity) of ERα neurons in the VMHvl leads to a behavior (mounting) that under natural conditions, is coupled with an overall decrease in activity. Further experiments examining the specific contribution of these neurons during sexual behavior are necessary to resolve this discrepancy.

### The hypothalamus and aggressive motivation

**Figure 6:**
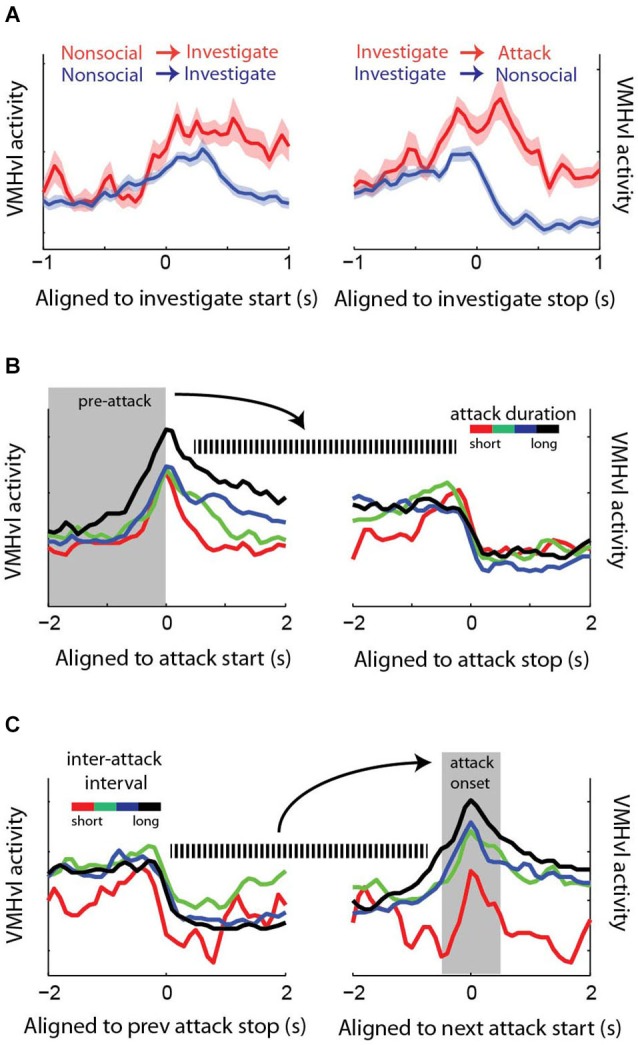
**Neuronal activity in the VMHvl is predictive of past and future aggressive events. (A)** Activity at the onset of a male investigative episode is increased if that episode leads to attack. **(B)** Activity prior to the start of the attack predicts the duration of the subsequent attack. **(C)** The inter-attack interval (i.e., time elapsed from the last attack) inversely correlates with activity at the onset of the next attack. (adapted from Falkner et al., 2014 with permissions).

Aggressive motivation can be loosely defined as the internal state that drives animals to seek out opportunities to perform aggressive actions. Signals in the brain that convey information about aggressive motivation should precede and perhaps predict future aggressive actions. Recordings of VMHvl neurons during intermale aggression revealed that VMHvl activity appears to carry information regarding past, current and future attack events (Falkner et al., 2014). First, neurons that respond during the investigation of a male intruder have an increased response if that investigative episode transitions to an attack compared to the response during investigation if the animal turns and walks away without attack (Figure 6A). Second, ramping activity prior to the onset of an attack in VMHvl neurons predicts duration of the subsequent attack: higher activity during the pre-attack period correlates with longer attacks (Figure 6B). Next, the time elapsed from the previous attack (the inter-attack interval) can predict activity of the onset of the next attack: the longer the wait from the last attack, the larger the attack response is for the next attack (Figure 6C). Furthermore, VMHvl activity is negatively correlated with latency to the next attack even when the animal’s proximity to odor cues and motion are controlled for. Lastly, increased VMHvl activity towards male intruder or male urine is sustained after the stimulus source is removed (Falkner et al., 2014). Thus, VMHvl neurons convey not only a rich variety of conspecific sensory cues, but also seem to predict aspects of future aggression, consistent with a role for the VMHvl in aggressive motivation.

Stimulation-evoked behaviors can also be interpreted through the lens of aggressive motivation. Early electric stimulation experiments showed that hypothalamic stimulation promotes not only attack but also approach towards a potential attack target (Roberts and Kiess, 1964). A common motivational mechanism that supports seeking behavior for both aggressive and sexual encounters could potentially resolve the apparent conflict between the observation that both sexual and aggressive actions can be evoked by stimulation and evidence from *in vivo* recordings which shows that VMHvl activity increases during aggression and decreases for sexual behaviors. If stimulation acts by increasing levels of motivated approach behavior, the end action may be determined by the sensory information or the specifics of the social interaction.

While these results seems to suggest a hypothalamic substrate for aggressive seeking behavior, it is difficult to assess the relationship between hypothalamic activity, approach behavior, and the social target being approached, because sensory stimuli are constantly changing during approach behavior and these changes may affect ongoing neural activity beyond the effects of the stimulation. These confounds necessitate the adoption and adaptation of new methods to assay social motivation. In many classic neuroscience experiments, motivation is assayed using standard operant response paradigms. In these paradigms, a reward (in most cases food or drink) is paired with an initially neutral motor response such as a nosepoke or lever press. By tuning the schedule of the reward delivery, the amount of work an animal is willing to expend to acquire the reward can be assayed, and this “work” reflects the motivation of the animal for the given reward. These paradigms have been commonly adapted to assay the motivation to acquire food, the motivational state that we call “hunger”, and several studies have found a clear link between hypothalamic activity and hunger. Most compellingly, optogenetic activation of agouti-related peptide (AGRP) neurons in the arcuate nucleus of the hypothalamus not only induces eating when food is readily available, but also promotes an increase in bar pressing to obtain food that is not immediately present (Krashes et al., 2011). In this way, an operant response can be used to disentangle the motivational state from the sensory cues associated from the reward itself.

Operant response paradigms have been applied thus far in a limited way to assess various types of social motivation including sexual (Everitt and Stacey, 1987) and maternal (Lee et al., 2000) motivation. Is there an equivalent motivational state for aggression? While we have no word in the English language like hunger to encapsulate this motivational state, aggression clearly exhibits several behavioral and neural features associated with other motivated behaviors such as feeding and sex. For example, motivated behaviors typically exhibit satiation, meaning that repeated consummatory actions (e.g., feeding or mating) reduce the underlying motivation to seek out further opportunities to perform these specific actions. Aggressive individuals across several species exhibit satiety after repeated attacks, exhibiting decreasing number of initiated attacks over time, and then exhibit an appetitive rebound after a prolonged non-aggressive interval (Connor, 1974), with some species exhibiting persistent levels of satiety (Potegal, 1984). Additionally, similar to its role in other motivated behaviors, dopamine appears to play a central role in aggression (Wise, 2004; de Almeida et al., 2005). When animals anticipate a fighting opportunity, dopamine levels increase (Ferrari et al., 2003). Antagonizing dopamine receptor in the nucleus accumbens reduces natural aggression, while lesions of the ventral tegmental area (VTA), a harbor for dopaminergic cells, blocks the HAA stimulation-evoked attack in rats (Proshansky et al., 1974; Couppis and Kennedy, 2008). Finally, a handful of studies have employed operant response paradigms to demonstrate that the opportunity to attack can be rewarding for certain individuals (Turnbough and Lloyd, 1973; Fish et al., 2002, 2005; Couppis and Kennedy, 2008; May and Kennedy, 2009). Aggressive mice can readily learn the association between the operant response and the introduction of a weaker intruder that serves as the “reward”. In these studies, fixed interval and fixed ratio schedules have been employed to assess the motivation for a single aggressive event. The operationalization of aggressive interaction is a critical next step for assessing the relationship between hypothalamic activity and aggressive motivation, since these paradigms allow for better control of sensory and motor variables and may distinguish the motivated state from the aggressive action.

### Towards a computational framework for hypothalamic functioning

While new techniques for functional manipulation and recording have deepened our understanding of the role of the hypothalamus in aggression, we are still far from a complete quantitative framework. However, our knowledge about evoked behaviors and types of signals that drive neurons in the VMHvl allows us to speculate about potential models for aggression. One possible model is that the VMHvl acts by performing a sensorimotor transformation, relaying a behaviorally relevant motor command through the transformation of specific sensory signals.

How could this transformation be implemented? Decades of neurophysiological recordings from defined cortical and subcortical circuits provide a useful model for understanding these computations. The oculomotor system is perhaps the canonical circuit described as carrying out a sensorimotor transformation. Sensory information in the form of visual stimuli can be used to guide specific motor sequences, in this case, saccadic eye movements. Neurons at the input level of this circuit can respond to purely sensory (visual) information, and neurons at the output level signal the upcoming movement. In this sensorimotor circuit, sensory information is mapped from visual coordinates into saccade coordinates by neurons in the parietal cortex, superior colliculus, and other key structures that contain neural representations of either the visual stimulus, the saccadic location, or both, and are subject to constant updating by changing environmental factors (Colby and Goldberg, 1999; Optican, 2005). Importantly, neurons in the oculomotor system can signal the location of visual information that is no longer present, and this “persistent” activity can connect sensory representations and motor signals that are separated in time.

**Figure 7:**
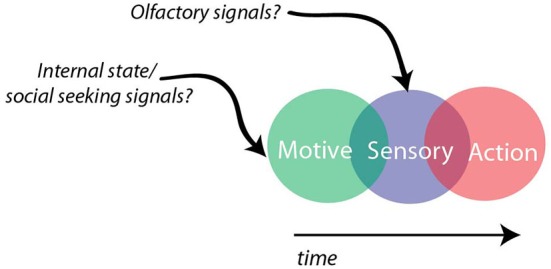
**Sensorimotor transformation as a possible model for hypothalamic function**. VMHvl neurons have overlapping sensory, action, and sensory-action preferring neurons that may be influenced by social specific olfactory and internal motivational signals.

Hypothalamic circuitry for aggression shares some broad similarities to other sensorimotor transformation circuits. Neurons in the VMHvl can preferentially signal information about the sensory environment (e.g., olfactory cues from male and female conspecifics) and also can preferentially signal the future actions that are coupled with these cues (e.g., attack or mating). In addition, neurons that signal both sensory and action related information (green dots Figure 4D) could serve to bridge sensory and motor representations within the VMHvl. However, since olfactory sensory information is encoded at a different level of complexity relative to the visual system, future computational models will need to account for these fundamental differences. In addition to being supported by current physiological characterizations, the anatomical architecture of the VMHvl could potentially support such a computation. The VMHvl, a densely glutamatergic subnucleus, has a high degree of recurrent connectivity that could support the persistence of a sensory or extrasensory signal (Nishizuka and Pfaff, 1989; Choi et al., 2005). If the role of the VMHvl is to carry out a sensorimotor transformation, one testable hypothesis would be that neurons carrying more action-related information may preferentially project to premotor structures, while neurons carrying more sensory-related information will be preferentially targeted by sensory relays (e.g., medial amygdala) and project more locally, allowing that information to persist within the VMHvl microcircuit. In future studies, this hypothesis and others could be tested by combining projection specific tagging and electrophysiology to compare electrophysiological responses with connectivity (Jennings et al., 2013). Moreover, future studies on aggressive motivation may reveal discrete or overlapping neuronal populations within the hypothalamus that may serve to combine motivational signals with incoming sensory information to drive aggressive actions (Figure 7).

## The aggression circuit beyond VMHvl

Of course, the hypothalamus does not act alone to elicit aggression. In rats, the efferent connections from hypothalamic aggression related cells have been examined by comparing anterograde tracing patterns from putative HAA and its adjacent “hypothalamic grooming area (HGA)” and by examining HAA stimulation of induced IEG expression or deoxyglucose uptake pattern (Roeling et al., 1993, 1994; Roberts and Nagel, 1996; Halasz et al., 2002b). These techniques revealed candidate regions that may be recruited during hypothalamic stimulation, many of which were also activated during stimulation of non-attack related areas. This overlap is not surprising, as the HAA covers multiple hypothalamic nuclei with heterogeneous connectivity patterns, and delineations between anatomically and functionally identified subregions of the hypothalamus are unclear.

The recent identification of ERα/PR cells in the VMHvl as a key population for aggression offers an opportunity to track other components in the circuit genetically (Yang et al., 2013; Lee et al., 2014). Yang et al. (2013) used lentivirus to express alkaline phosphatase (PLAP) which labels neuronal processes of PR-expressing cells in the VMHvl and observed a refreshingly simple projection pattern: PLAP+ axons were found in the anteroventral periventricular hypothalamic nucleus (AVPV), preoptic area (POA) and periaqueductal gray (PAG). Interestingly, the PLAP+ axons in AVPV are sexually dimorphic: the fiber density in females is 7-folder greater than the density in males. While unexpected, this difference may be related to the well-known role of VMHvl in lordosis (Pfaff and Sakuma, 1979a,b). Other previously identified VMHvl downstream targets including the medial amygdala (MEA) and bed nucleus of stria terminalis (BNST) didn’t appear to be targeted appreciably by the PLAP+ axons (Yang et al., 2013), suggesting that aggression-related cells may selectively project to a subset of VMHvl downstream regions. In rat, stimulation of PAG can also elicit attack although the responses are sometimes accompanied by severe motor disturbance (Mos et al., 1982). Though POA is mostly known for its role in male sexual behaviors, electric lesion of the area suppresses both sexual and aggressive behaviors in rats without affecting animals’ predatory mouse killing behavior (Bermond, 1982; Albert et al., 1986a,b). Similar to the VMHvl, both PAG and POA are enriched in hormone receptors such as ERα and androgen receptor (AR), which have profound influence on social behaviors (Murphy et al., 1999; Merchenthaler et al., 2004). Future studies using optogenetic tools to achieve pathway specific functional manipulation will help elucidate the roles of VMHvl → POA and VMHvl → PAG projections in VMHvl evoked aggression (Tye and Deisseroth, 2012).

## Concluding remarks

Aggression, which has evolved to resolve competition and secure resources, is an essential part of many species’ ethological repertoire. However, disregulated or pathological aggression poses huge risks to society. Although aggression studies thrived in the early and middle 20th century, this line of research has substantially declined since then, especially in comparison to other innate and “emotional” behaviors such as fear (Blanchard et al., 2003). The reasons for the decline are complicated and include issues related to both animal and social welfare (Blanchard et al., 2003; Anderson, 2012). Efforts to translate research advances in aggression into clinical use during the 1950s and 1960s have a sordid history, as prominent researchers of the day advocated the use of brain stimulation techniques and psychosurgery on prisoners and as a solution to race-charged problems such as urban rioting and political protest (Mark et al., 1968; Breggin, 1975a,b). At least in part because of this, research on aggression fell out of fashion over the next few decades.

In addition to the political over-interpretation of aggression studies beyond the line of basic research, technical limitations also obstructed advances in the field. As mentioned, classic lesion and stimulation methods lack adequate spatial resolution to pinpoint aggression-relevant cells. However, the recent emergence of genetically based functional manipulation and tracing methods allows one to target neurons with specific functional relevance, projection patterns or molecular features, greatly improving precision in cell manipulation (Lima et al., 2009; Cardin et al., 2010; Yizhar et al., 2011; Madisen et al., 2012; Osakada et al., 2012; Tye and Deisseroth, 2012; Guenthner et al., 2013; Kawashima et al., 2013). Moreover, miniaturized *in vivo* electrophysiological recording devices, wired or wireless, and deep brain optical measuring devices make it possible to follow cell activity reliably over a long period (Fan et al., 2011; Szuts et al., 2011; Cui et al., 2013; Ziv et al., 2013). Using these novel approaches, breakthroughs in research in animals may lead to a greater understanding of pathological violence and potentially to more humane treatment strategies.

## Conflict of interest statement

The authors declare that the research was conducted in the absence of any commercial or financial relationships that could be construed as a potential conflict of interest.
